# Next-generation cancer vaccines: targeting cryptic and non-canonical antigens for precision immunotherapy

**DOI:** 10.37349/etat.2025.1002338

**Published:** 2025-09-25

**Authors:** Anu Shibi Anilkumar, Sheena Mariam Thomas, Ramakrishnan Veerabathiran

**Affiliations:** Weill Cornell Medicine, USA; Human Cytogenetics and Genomics Laboratory, Faculty of Allied Health Sciences, Chettinad Hospital and Research Institute, Chettinad Academy of Research and Education, Kelambakkam 603103, Tamil Nadu, India

**Keywords:** cancer, immunotherapy, vaccines, canonical antigens, cryptic antigens

## Abstract

Cancer immunotherapy has revolutionized oncology by harnessing the immune system to target tumor cells. Cancer vaccines that trigger immune responses specific to tumors are becoming more and more popular among new approaches. Nevertheless, traditional tumour-associated antigens are susceptible to immune tolerance and frequently show low immunogenicity. The revolutionary potential of cryptic and non-canonical antigens as new targets for precision immunotherapy is examined in this review. Due to their enhanced tumor selectivity and ability to evade central tolerance, these unconventional antigens present encouraging options for vaccine development. This review examines the mechanisms underlying their antigen production, advanced technologies for their discovery, and various vaccine platforms, highlighting their potential to drive the next generation of cancer vaccines.

## Introduction

Cancer refers to a collection of diseases marked by the uncontrolled growth of abnormal cells, which may invade or spread to other areas of the body [1]. Although substantial progress has been made in cancer research and treatment, it continues to pose a major global health challenge and remains one of the leading causes of death. Early detection and timely treatment have greatly enhanced survival outcomes [2]. Cancer continues to hinder improvements in life expectancy worldwide [3]. As reported by the World Health Organization (WHO), cancer was responsible for around 10 million deaths in 2020, representing roughly one in six deaths globally, and is expected to cause 16.2 million deaths and 28 million new cases each year by 2040 [4, 5].

Over the past 170 years, milestones such as the development of anesthesia, X-rays, chemotherapy, immunotherapy, and gene therapy have markedly improved cancer management and post-treatment results [5]. Among these, immunotherapy represents a revolutionary shift in treatment strategies, aiming to augment the immune system’s capacity to recognize and eliminate tumour cells [6]. Unlike traditional therapies, immunotherapy reactivates immune responses using immune cells, cytokines, and chemokines, thereby modifying the tumour microenvironment (TME), boosting antitumour activity, and reducing recurrence risk [7].

Specifically, immune checkpoint inhibitors and CAR T-cell therapies have been recognized as potent FDA-approved immunotherapies that activate the immune system to target and destroy cancer cells [8]. However, not all cancers or patients respond uniformly to these treatments. Over the past decade, immunotherapy has been further transformed by checkpoint inhibitors, which harness the immune system’s natural capacity to differentiate between the body’s cells and foreign invaders, offering a broadly applicable strategy across multiple tumour types regardless of their molecular growth pathways [9].

Personalized immunotherapy has shown promise in patients without actionable genetic mutations or those who develop resistance to conventional chemotherapy, providing alternative strategies [10]. However, cancer vaccines are designed to activate the immune system by presenting it with tumour-specific antigens (TSAs), and hold immense therapeutic and preventive promise, but their efficacy is hindered by various challenges.

Vaccine efficacy is hindered by the immunosuppressive characteristics of the TME, genetic instability, and tumour heterogeneity. Overcoming these challenges by focusing on particular elements of the TME, such as immune checkpoints or fibroblast activation, may lead to better treatment outcomes [11]. Cancer vaccines typically present tumour antigens in forms such as whole cells, proteins, peptides, RNA, or DNA, and are often combined with adjuvants like dendritic cells (DCs), CD40 ligand, oil-water emulsions, or Toll-like receptor (TLR) agonists to boost immune responses [7].

Vaccines have made unparalleled contributions to public health, and recent technological advancements have opened the door to antigen-specific cancer vaccines. Progress in tumour immunology, messenger RNA (mRNA) platforms, lipid nanoparticles, next-generation sequencing, artificial intelligence (AI), and innovative clinical trial designs, especially when combined with checkpoint inhibitors, is driving their growing success [12]. New technologies like AI, particularly in the areas of immunogen production and antigen selection, have emerged as a potent instrument that greatly simplifies traditional procedures. During the COVID-19 pandemic, AI-driven algorithms made it possible to quickly identify new antigens, which sped up the creation of vaccines to a level never seen before. Through the precise and effective evaluation of immunogenicity and the correct prediction of antigenic epitopes, machine learning methods such as random forest and deep learning have significantly improved this procedure [13].

According to Burnet’s immune surveillance theory, the immune system continuously detects and eliminates nascent cancer cells [14]. In line with this, recent research highlights cryptic antigens, which are unconventional translation products presented on major histocompatibility complex class I (MHC-I) molecules. These arise when eukaryotic ribosomes translate regions beyond the main open reading frame (ORF), generating peptides that can serve as novel antigens [15]. Research has demonstrated that cryptic epitopes are capable of triggering antiviral immune responses, indicating their potential to enhance vaccine efficacy [16, 17].

Closely related are non-canonical MHC-associated peptides (ncMAPs), which are derived from aberrant translation of small ORFs (sORFs) in typically non-protein-coding regions like untranslated regions (UTRs), long non-coding RNAs (lncRNAs), and pseudogenes. These non-canonical antigens have demonstrated the ability to provoke antitumour immune responses and are now being explored across various cancer types [18]. The recurrence of non-canonical peptides across multiple cancer types offers compelling opportunities for the development of universal or semi-universal cancer vaccines [19].

With an emphasis on their potential to advance precision immunotherapy, this review examines the field of cryptic and non-canonical antigens, exploring their sources, mechanisms, detection techniques, vaccine development strategies, and related challenges.

## Cancer antigens

### Tumour-associated vs. TSAs

Tumour antigens are generally divided into two main categories: tumour-associated antigens (TAAs) and TSAs. TAAs are highly expressed in tumour cells but may also be present at lower levels in normal tissues [20, 21]. This group includes differentiation antigens (such as melanocyte antigens), viral antigens (e.g., HPV), mutated proteins (such as p53), overexpressed proteins (like HER2), and cancer/testis (CT) antigens like MAGE and NY-ESO-1, which are typically confined to germ cells [22]. Although TAAs are not entirely tumour-specific, their elevated expression in malignant cells can trigger cytotoxic T lymphocyte (CTL) responses [23]. However, due to their presence in normal tissue, TAAs often induce immune tolerance and are less immunogenic [24–26].

T cells recognize peptide-MHC complexes rather than surface proteins, so vaccine targets do not need to be membrane-bound. Co-stimulatory signals are also required for full T-cell activation. Identifying lipid antigens and stroma-associated targets may further expand therapeutic options [27]. In contrast, TSAs, particularly neoantigens, arise from tumour-specific somatic mutations, including SNVs, indels, frameshifts, fusion genes, and structural variants, and are absent in normal tissues [23]. Because they are truly non-self, neoantigens are highly immunogenic, although they are often patient-specific [21]. Some tumour-specific peptides may result from phosphorylation or abnormal splicing, and if they are occasionally expressed at low levels in normal tissue, they may not qualify as true neoantigens. Thus, the term “neoantigen” should be used only when exclusive tumour-specific expression is confirmed [28]. Tissue-specific antigens, another subgroup, are shared with the tissue of origin and are sometimes expressed only during certain differentiation stages [29].

Because CD8^+^ effector T lymphocytes can directly destroy cancer cells, they are important mediators of tumor-specific immunity. However, tumor-specific CD4^+^ T helper cells are necessary for the activation and persistence of these CD8^+^ T cells, and their presence is necessary for their effective operation. Techniques to increase CD4^+^ T cell activation have been developed to improve this process. Tumor cells can directly present tumor-derived peptides to CD4^+^ T helper lymphocytes when they have MHC-II molecules on them. By removing the requirement for soluble tumor antigens and host antigen-presenting cells (APCs), this direct presentation strengthens the immune response against tumors [30].

Tumors, including MUC1 and HER2, exhibit abnormal expression of TAAs. DCs [especially conventional DC1 (cDC1) subsets] frequently cross-present TAAs, which are processed and presented via MHC-I to CD8⁺ T cells and MHC-II to CD4⁺ T helper cells. Due to self-tolerance, the immune response to TAAs is often less strong than that to neoantigens; however, successful TAA targeting can trigger strong Th1 responses (IL-2, IFN-γ, and TNF-α), which encourage the activation of CTL and NK cells. While Tregs inhibit anti-tumor immunity through IL-10 and TGF-β, a dominant Th2 response (IL-4, IL-10) is associated with tumor growth. In TAA-specific responses, a low Th1/Th2 ratio is linked to a poor prognosis [31].

### Canonical antigens

The standard protein-coding region of an mRNA is identified as the most extended ORF beginning with an AUG start codon and is usually subject to evolutionary pressure. Processes such as alternative splicing and polyadenylation can produce mRNA isoforms with different coding sequences. Genes that do not contain a long and/or conserved ORF are typically categorized as lncRNAs or pseudogenes, based on the idea that longer proteins are more likely to adopt stable structures with functional biological roles [29].

Neoantigens are processed by the endogenous system so that they are presented on MHC-I molecules, mainly by human leukocyte antigen (HLA)-A, -B, and -C alleles to CD8⁺ CTLs. Because these antigens are not susceptible to central tolerance, they elicit potent CTL responses that facilitate the destruction of tumor cells, making them extremely immunogenic. Although Th2 responses can occur and are linked to humoral immunity, this antigen class usually elicits Th1-biased immune responses, which are typified by the production of IFN-γ and IL-2 [32, 33].

In recent years, mass spectrometry (MS)-based immunopeptidomics, in combination with novel proteogenomic techniques, has enabled the identification of novel canonical and non-canonical cancer-specific antigens. These antigens arise due to genetic and epigenetic changes that take place during cancer development, affecting the cellular transcriptome, translatome, proteome, and antigen presentation systems [34]. Identifying and categorizing immunogenic epitopes is vital for advancing cancer vaccine strategies and adoptive T cell-based immunotherapies. Notably, neoantigen peptides originating from mutated proteins are of special interest because they are specific to tumours and often unique to individual patients, significantly contributing to the success of checkpoint blockade immunotherapy [35]. The key features distinguishing canonical, cryptic, and non-canonical antigen types in tumour immunology are tabulated in Table 1.

**Table 1:** Key features distinguishing canonical, cryptic, and non-canonical antigen types in tumour immunology.

**Feature**	**Canonical**	**Cryptic**	**Non-canonical**
Origin	Annotated ORFs	Unannotated regions	Non-coding or shifted ORFs
Start codon	AUG	Near-cognate codons	AUG/Near-cognate
Stability	Stable	Often unstable	Unstable/Short-lived
Immunogenicity	Moderate	High	High
Tumour specificity	Variable	High	High
Detection methods	MS, WES, RNA-seq	Immunopeptidomics	Ribo-seq, MS

MS: mass spectrometry; ORFs: open reading frames; Ribo-seq: ribosome profiling; RNA-seq: RNA sequencing; WES: whole exome sequencing.

### Cryptic antigens

The term cryptic antigens refers to “hidden” or “invisible” peptides that arise from genomic loci not previously annotated or studied. These peptides can be generated through translation of both non-canonical ORFs (ncORFs) and unannotated ORFs [36]. Relatively short ORFs typically encode cryptic proteins and often begin translation with non-AUG near-cognate codons (most of which, except for CUG, are decoded as methionine). Cryptic proteins are identified in the immunopeptidome far more often than canonical proteins, indicating their significant role in antigen presentation [37].

These antigens are displayed on MHC-I molecules and cause strong CTL responses when they are exposed in malignancies as a result of abnormal transcription or translation. Recent research on pancreatic cancer showed that the tumor immunopeptidome contains a large number of cryptic antigens and that specific peptides can directly kill tumor cells by triggering cytotoxic T-cell responses. Significant anti-tumor activity was also demonstrated by experimental vaccination using cryptic peptide pools in preclinical models [38].

Over the past decade, genomic-based approaches combined with immunopeptidomics have uncovered the presence of nonmutated peptides originating from regions previously thought to be non-coding. These cryptic or non-canonical peptides, found outside conventional coding exon boundaries, are gaining attention for their tumour-specific expression and recurrent appearance across cancer patients. Their nonmutated status makes them attractive targets for cancer immunotherapy [39].

### Non-canonical antigens

Non-canonical antigens originate from unconventional regions of the genome, such as alternative ORFs, introns, UTRs, lncRNAs, and pseudogenes. These regions have historically been overlooked due to the difficulty in detecting actively translated ORFs. The vast number of potential ORFs in a eukaryotic genome, reaching into the millions, contributes to the overall complexity [29]. Recent progress in proteomics and ribosome profiling (Ribo-seq) has uncovered a previously unrecognized array of non-canonical proteins encoded by nontraditional ORFs that lie beyond standard gene annotations [40].

Significantly, non-canonical proteins can originate not only from regions thought to be non-coding but also from the +1 or +2 reading frames of canonical ORFs. Far from being mere byproducts of random translation, many of these proteins carry out essential and varied cellular roles. In contrast to traditional ORFs, ncORFs are generally shorter, exhibit lower levels of transcription and translation, often initiate at near-cognate start codons, and display reduced stability within living cells [41–43].

Though often shown by MHC-I molecules on tumor cells, ncMAPs are highly immunogenic due to their lack of central tolerance. MS and prediction techniques like network-based MHC peptide binding prediction (NetMHCpan) have found several ncMAPs in malignancies like acute myeloid leukemia, and some of these have been demonstrated to trigger potent CTL responses that can destroy tumor organoids ex vivo [18]. It is shown that in murine cancer cell lines and human primary tumours with diverse haplotypes, approximately 90% of TAAs were derived from non-canonical genomic regions [44]. This non-canonical neoantigen landscape, often termed the “dark” side of the genome, plays a substantial role in shaping the immunogenic properties of tumours [44].

## Mechanisms of antigen emergence and detection

### RNA splicing variants

In eukaryotic genes, the protein-coding regions are interspersed with non-coding introns, which are excised from the precursor mRNA (pre-mRNA) during processing, and the remaining coding regions, known as exons, are joined to form mature mRNAs in a fundamental process called pre-mRNA splicing [45]. Mutations affecting canonical splice sites can impair this process, leading to gene dysfunction and contributing to disease. These splice site mutations, often detected in clinical diagnostic settings, are estimated to account for about 10% of all pathogenic mutations. Although primarily affecting the major spliceosome, which handles most introns, similar disruptions have been reported in the minor spliceosome as well [46].

Over 90% of human protein-coding genes experience alternative splicing [36]. In cancer, the splicing machinery is frequently dysregulated, resulting in tumour-specific isoforms that influence cell proliferation, motility, and drug response [47, 48]. Abnormal splicing events can impact almost every facet of tumour biology, such as cell cycle control, metabolism, invasion, formation of new blood vessels (angiogenesis), metastasis, and programmed cell death [49].

Alternative transcripts generated through cryptic or non-canonical splice sites can give rise to abnormal proteins, which may serve as cancer biomarkers. These alterations can result in exon skipping or the production of truncated or dysfunctional proteins [50]. Because of their tumour specificity, such transcripts are increasingly explored as diagnostic markers and therapeutic targets. Current strategies to modulate splicing include the use of small molecules and splice-switching antisense oligonucleotides (SSOs) [51].

### Post-translational modifications (PTMs)

PTMs are covalent modifications to proteins, such as proteolytic cleavage or the addition of chemical groups like acetyl, phosphoryl, glycosyl, and methyl moieties. These changes influence protein structure, dynamics, and function, playing critical roles in various biological processes. PTMs may be reversible or irreversible, typically regulated by enzymes, although they can also arise from aging or chemically reactive environments [52, 53]. PTM patterns can shift in response to infection, inflammation, transformation, or cellular stress. Some modifications persist during antigen processing, resulting in the presentation of altered peptides by MHC-I or MHC-II molecules. These modified epitopes are recognized by both humoral and cellular immune components, broadening the antigenic repertoire [54].

**Figure 1:**
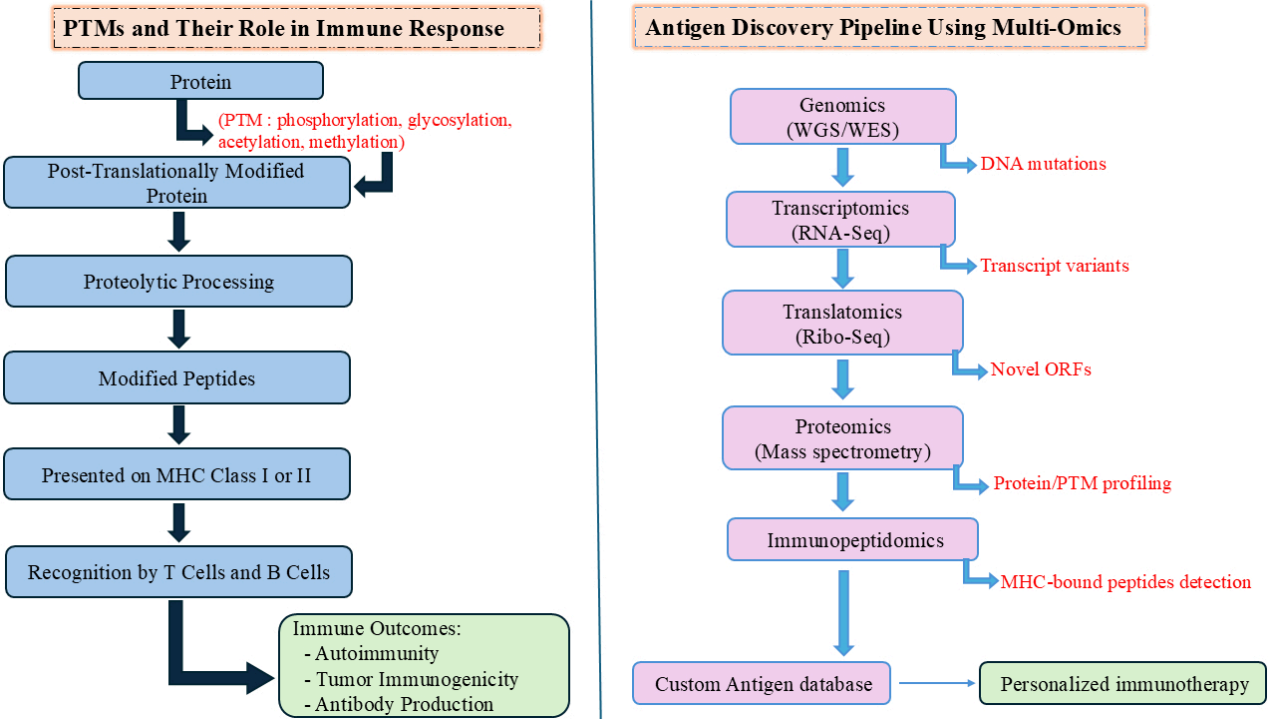
**Post-translational modifications (PTMs) and multi-omics antigen discovery pipeline.** The diagram illustrates PTMs like phosphorylation, glycosylation, acetylation, and methylation alter proteins post-translation, followed by proteolytic processing into modified peptides. These peptides are presented by MHC-I or MHC-II molecules and recognized by T and B cells, influencing immune responses. Multi-omics integration in antigen discovery involves sequential analysis: genomics for DNA mutations, transcriptomics for transcript variants, translatomics for novel ORFs, proteomics for protein and PTM profiling, and immunopeptidomics for MHC-bound peptide detection. Outputs feed into a custom antigen database to design personalized immunotherapies. MHC: major histocompatibility complex; WGS: whole genome sequencing; WES: whole exome sequencing; RNA-seq: RNA sequencing; Ribo-seq: ribosome profiling; ORFs: open reading frames.

PTM-containing peptides are increasingly linked to autoimmune diseases and cancer. In TAAs, PTMs may enhance immunogenicity by disrupting tolerance or mimicking non-self structures. Notably, phosphorylated TAAs are often more effectively presented by HLAs and can trigger immune responses, including autoantibody production, making them promising targets for cancer immunotherapy [55]. Recent advances in MS-based proteomics, chemical biology, fluorescence assays, and bioinformatics have greatly improved the detection and characterization of PTM sites, underscoring their relevance in disease research and immunotherapeutic development [56]. The PTMs and multi-omics antigen discovery pipeline is depicted in Figure 1.

### Proteasome splicing and peptide fusions

The immune system continuously surveys cellular health to prevent tumour development and combat viral infections. CD8⁺ CTLs are key players in this process, identifying and eliminating cells that display abnormal peptides on MHC-I molecules. These peptides are mainly produced by the proteasome, a major proteolytic complex that breaks down proteins within the cell. The proteasome exhibits three main catalytic activities: caspase-like, chymotrypsin-like, and trypsin-like, which cleave after acidic, hydrophobic, and basic residues, respectively [57].

Although the majority of peptides produced by the proteasome are broken down further for amino acid reuse, a specific subset is transported into the endoplasmic reticulum (ER) by the transporter associated with antigen presentation (TAP). Within the ER, these peptides bind to newly formed HLA-I molecules, which are then delivered to the cell surface to be recognized by CD8⁺ T cells [58].

In addition to proteolysis, both standard proteasomes (SP) and immunoproteasomes (IP) can generate neo-epitopes through a mechanism called proteasome-catalyzed peptide splicing (PCPS), where noncontiguous peptide fragments from the same antigen are ligated to form novel sequences. These spliced peptides can also serve as MHC-I ligands and be exhibited on the cell surface [59]. Importantly, PCPS is not a random process; it prefers specific peptide motifs distinct from those used in standard hydrolysis, resulting in a unique set of epitopes with differing HLA-binding affinities [60]. This may explain why some self-antigens are presented exclusively as spliced peptides.

To improve vaccine efficacy, especially against rapidly mutating intracellular pathogens, it is essential to design vaccines that elicit strong CD8⁺ T cell responses. Recombinant vaccine vectors encoding minor pathogen-derived antigens are being evaluated for their protective potential. However, the high polymorphism of HLA molecules complicates the prediction of universally effective epitopes. Although in silico tools are used to identify candidate peptides, they often overlook spliced epitopes. Including PCPS-derived peptides in antigen selection could broaden the pool of immunologically relevant targets, enhancing the robustness and population coverage of the immune response [61].

### Translation from non-coding regions and upstream ORFs

ncORFs have emerged as a significant source of proteomic diversity, arising from regions such as overlapping downstream ORFs (odORFs), overlapping upstream ORFs (ouORFs), upstream ORFs (uORFs), internal ORFs (intORFs), lncRNAs, and downstream ORFs (dORFs). These ncORFs challenge traditional coding paradigms and expand the proteome beyond canonical annotations. Their translation is enabled through mechanisms such as leaky ribosomal scanning (in the case of uORFs) and the use of alternative initiation codons. Despite limited evolutionary conservation, many ncORFs have been linked to trait-associated genetic variants and play critical roles in disease and cancer biology. Notably, ncORFs serve as a significant source of TSAs, underscoring their immunological relevance [62].

Among ncORFs, an increasing number of sORFs have been identified, particularly within non-coding RNAs, encoding microproteins typically under 100 amino acids. These microproteins are often aberrantly expressed in cancers and contribute to tumour initiation and progression. Depending on the context, they may exhibit oncogenic or tumour-suppressive functions, positioning them as promising diagnostic or prognostic biomarkers and potential therapeutic targets. Their dysregulation is frequently associated with alterations in RNA stability, translation efficiency, or protein turnover, often driven by disrupted transcription factor networks [63]. Although lncRNAs were traditionally classified as non-coding due to the absence of AUG-initiated ORFs exceeding 100 amino acids, it is now evident that many lncRNAs do harbor peptide-coding ORFs. These overlooked regions can give rise to functional microproteins with roles in cellular regulation and disease [64].

### Technologies for antigen discovery

The use of integrated multi-omics approaches has dramatically enhanced the identification of TAAs. Cancer-specific changes at the genomic, transcriptomic, and translational levels give rise to novel protein sequences, which serve as a valuable source of TSAs. A prominent method in this field is proteogenomics, which combines DNA-based techniques such as whole exome sequencing (WES) and whole genome sequencing (WGS) with RNA sequencing (RNA-seq) and Ribo-seq, alongside MS-based proteomics and immunopeptidomics [65].

This strategy supports the development of tailored protein databases that go beyond conventional references by incorporating predicted novel sequences from WES, WGS, RNA-seq, or Ribo-seq. These customized databases enhance the detection of both canonical and non-canonical peptides using retention time (RT) and tandem MS/MS spectral data. Notably, immunopeptidomics often captures peptides derived from unstable protein products that may escape detection in standard proteomics.

Ribo-seq is particularly powerful in identifying actively translated ORFs. It captures ribosome-protected RNA fragments, offering nucleotide-level resolution of translation events. This enabled the identification of thousands of ORFs, including those from lncRNAs, UTRs, and novel short ORFs (< 100 amino acids), many of which had remained undetected by traditional annotation methods. A single Ribo-seq experiment can detect translation from ~ 11,000–12,000 genes, closely aligning with expressed mRNA profiles, and surpassing typical detection by MS. Interestingly, while many Ribo-seq-identified ORFs are not readily detected in standard shotgun proteomics, their products appear in immunopeptidomics datasets, supporting the notion that unstable or short-lived proteins may serve as essential sources of MHC-bound peptides and TSAs [65].

Alongside Ribo-seq, MS remains a cornerstone in antigen discovery. When coupled with liquid chromatography (LC), MS enables high-resolution analysis of complex antigens, including disulfide bonds, amino acid sequences, and PTMs. Among PTMs, glycosylation, particularly in viral glycoproteins, plays a vital role in shaping immune recognition. MS-based glycan profiling has been instrumental in the design and optimization of vaccines against HIV, influenza, dengue, Ebola, and other infectious agents, and it holds similar potential for cancer vaccine development [66].

Together, these technologies form the backbone of modern antigen discovery, enabling the identification of both canonical and cryptic targets for next-generation immunotherapies and personalized cancer vaccines.

## Immunogenicity and presentation of cryptic/non-canonical antigens

### Immunogenic properties compared to canonical antigens

A new era in cancer vaccine development is emerging, driven by more profound insights into immune responses and the expanding understanding of tumour-associated neoantigens. A significant advancement in this field is the application of MHC-I immunopeptidomics via MS, which allows the integration of antigen profiling with immunogenomics to identify and prioritize effective mutations for vaccine design. A large-scale analysis of immunopeptidomes from 486 samples across 26 published cancer studies using a harmonized approach led to the creation of a novel peptide catalog. This catalog encompassed peptides from both canonical sources, such as exonic regions and post-translationally modified proteins, and non-canonical sources, including intronic, frameshifted, or UTRs, revealing a broad spectrum of recurrent in-frame antigens and out-of-frame neoantigens [19].

The display of peptides by HLA molecules on the surface of cancer cells is crucial for T cell-driven immune surveillance. Understanding the antigenic landscape of tumours is therefore essential for developing effective immunotherapies. In this context, proteogenomics has been successfully utilized to provide a more comprehensive view of antigenic peptides derived from both coding and non-coding regions of the genome [41].

Conventional proteins, with a median length of around 400 amino acids, naturally generate more MAPs due to their larger size. In contrast, cryptic MAPs derived from non-canonical proteins exhibit three distinguishing characteristics: they are generally shorter, show different preferences for MHC allotype binding, and harbor a higher frequency of genomic polymorphisms. A marked enrichment of 8-mer peptides and a depletion of 10–11-mers in cryptic MAPs, compared to conventional MAPs, further suggests they undergo distinct peptidase processing. Unlike conventional MAPs, which are typically generated through proteasomal cleavage followed by N-terminal trimming, cryptic MAPs often require only the removal of the N-terminal methionine. The exceptionally short length of their source proteins implies that many cryptic MAPs may bypass proteasomal degradation altogether. Additionally, the differences in amino acid usage around their C-termini support the hypothesis that their processing could be proteasome-independent [67].

Additional differences in the biochemical and immunogenic properties of canonical and non-canonical peptides were highlighted in a study conducted by Cai et al. [18] (2024). This study reported that both peptide types displayed similar tissue distribution patterns, with each tissue contributing a unique peptide set. Sex organs contribute the highest number of unique sequences. The brain, owing to its cellular complexity and heterogeneity, showed the most incredible diversity in peptide expression. Moreover, non-canonical peptides were generally enriched in cysteine and tryptophan residues. Tryptophan, due to its hydrophobic nature and distinctive aromatic structure, plays a key role in enhancing antigen-antibody interactions and stabilizing epitope structures, thereby improving immune recognition and response attributes valuable for vaccine and diagnostic reagent development. In contrast, canonical peptides contained acidic residues such as aspartate and glutamate more frequently. Notably, the study also found that non-canonical peptides had more stable binding to HLA-B alleles than canonical peptides, indicating that HLA molecules are well-equipped to present non-canonical peptides through standard antigen presentation mechanisms [18].

### MHC-I and MHC-II presentation pathways

MHC-I molecules play a vital role in cancer immune surveillance by displaying peptides derived from cytosolic proteins on the cell surface, allowing T cells to recognize and eliminate tumour cells [68]. Traditionally, tumour antigens were discovered within annotated coding regions; however, emerging techniques such as combining antigen presentation analyses with Ribo-seq have identified microproteins as significant contributors to cryptic antigen pools. Non-canonical proteins, which are typically unstable and short-lived, have been found to produce MHC-I peptides more efficiently with each translation event. Although these cryptic antigens can be detected through MHC-I immunopeptidomics, their originating proteins are often overlooked in whole-cell proteomics due to their fleeting existence [68].

The elevated presence of ncORF-derived peptides in the MHC-I peptidome compared to the cellular proteome may result from the efficient presentation of rapidly degraded and short-lived proteins. The immunopeptidome selectively enriches peptides from less abundant proteins like those from ncORFs, offering a broader representation of intracellular proteins. Additionally, the intrinsic features of non-canonical proteins, such as short length, instability, and subcellular localization, further facilitate their processing and presentation via the MHC-I pathway [68]. Effective recognition of MAPs on cancer cells is essential for the success of T cell-based immunotherapy. Ideally, tumour antigens should be crucial for tumour survival, expressed on all malignant cells, and absent from normal tissues [17].

The MHC-I pathway relies on the ubiquitin-proteasome system to degrade endogenous proteins into peptides, particularly those with precise C-terminal ends suited for MHC-I binding. These peptides, usually ranging from 8 to 10 amino acids in length, are loaded onto MHC-I molecules for display to CD8⁺ T cells. This system processes not only conventional proteins but also mutant transcripts, viral proteins, and aberrant translation products, broadening the antigen repertoire. However, many cancers evade immune detection by downregulating MHC-I expression commonly seen in tumours such as NSCLC, breast, colorectal, and hepatocellular carcinomas. Furthermore, intratumoural heterogeneity (ITH) in MHC-I expression contributes to resistance against immune-based therapies [69]. MHC presentation of cryptic (non-canonical) and canonical antigens is depicted in Table 2.

**Table 2:** MHC presentation of cryptic (non-canonical) and canonical antigens.

**MHC presentation aspect**	**Canonical antigens**	**Cryptic (non-canonical) antigens**
Source of antigen	Derived from well-annotated coding regions (exons), stable, long-lived proteins.	Derived from non-coding regions, frameshifts, introns, UTRs, or aberrant translation, typically short-lived proteins.
Processing pathway (MHC-I)	Proteasomal degradation followed by TAP-mediated peptide transport to the ER generates typical 8–10 aa peptides.	May bypass classical proteasomal cleavage; often only require N-terminal methionine removal; favor 8-mers over 10-mers.
Binding to MHC molecules	Generally strong and predictable based on established binding motifs and allele preferences.	Cryptic antigens often bind uniquely, showing stable binding, especially to HLA-B alleles, and differ in terminal aa usage.
Presentation efficiency	Abundant and well-presented due to stable protein origins and conventional processing.	Enriched in MHC-I immunopeptidome despite low abundance in whole-cell proteome; efficient due to rapid degradation.
Immunological visibility	More likely to be recognized as “self” by the immune system, limiting T cell activation due to central tolerance.	Escaping central tolerance makes it more likely to be recognized as foreign, enabling strong immune responses.
Clinical relevance	Commonly targeted in traditional cancer vaccines, they have limited success due to immune tolerance and tumour escape mechanisms.	Emerging targets for personalized vaccines are promising due to their tumour-specificity and high immunogenicity.

MHC: major histocompatibility complex; TAP: transporter associated with antigen presentation; ER: endoplasmic reticulum; aa: amino acids; UTR: untranslated region; HLA: human leukocyte antigen.

### Avoidance of central tolerance

TSAs are exclusively found in tumour cells and are absent from normal tissues. Due to their restricted expression in tumours, TSAs bypass central tolerance during T-cell development, enabling them to provoke strong T-cell responses without causing autoimmunity. In contrast, TAAs are normal self-proteins that are overexpressed in cancer cells compared to healthy tissues. Although TAAs are not unique to individual patients and are often shared among various cancer types, making them appealing targets for broadly applicable immunotherapies, they are identified as “self” by the immune system and undergo central tolerance, which limits their ability to generate strong immune responses [70].

Historically, cancer vaccines targeting TAAs have shown limited clinical efficacy, primarily due to the immune system’s tolerance to these antigens. A central challenge in targeting TAAs and cancer germline antigens (CGAs) is overcoming this tolerance without provoking “on-target off-tumour” toxicity, wherein the immune system also attacks healthy tissues expressing the same antigens. The expression level of these epitopes further complicates this balance: low antigen abundance may fail to activate T cells effectively and may even induce tolerance, whereas high levels can increase the risk of autoimmunity, particularly when the target is a self-antigen. To address these challenges, novel vaccine delivery strategies and combination therapies have been developed to help bypass central tolerance mechanisms and enhance the clinical efficacy of TAA-targeted immunotherapies [70]. Immunologic tolerance is defined as the selective lack of an immune response to particular antigens, while the immune system continues to respond, usually to other antigens [71].

## Next-generation vaccine platforms

### Peptide-based vaccines

Inactivated or attenuated pathogens naturally stimulate robust immune responses by presenting B- and T-cell epitopes in a conformation mimicking the native pathogen. However, subunit vaccines composed primarily of peptides or proteins often exhibit limited immunogenicity and may require multiple doses to achieve comparable immune activation. To enhance their efficacy, various strategies have been employed, such as multimeric epitope presentation using virus-like particles (VLPs) or nanoparticles, and incorporation of immunostimulatory adjuvants [72].

Cancer vaccines aim to stimulate the immune system to identify and destroy tumor cells. Peptide-based vaccines, in particular, use synthetic peptides, usually 20 to 30 amino acids long, that represent immunogenic epitopes from TSAs. These vaccines provide several benefits, including a favourable safety profile, ease of manufacturing, and demonstrated effectiveness in eliciting T-cell responses, as supported by multiple clinical studies. Nevertheless, enhancing their immunogenic potential remains a key challenge. For effective antitumour immunity, such vaccines must include epitopes capable of activating both CD8⁺ CTLs (via cross-presentation) and CD4⁺ helper T cells, which support CTL functions. The peptide sequence length is critical to ensure efficient engagement of both T cell types [73].

Peptide-based vaccines are commonly developed in two main formats: short peptides and synthetic long peptides (SLPs). Short peptides, usually ranging from 8 to 12 amino acids in length, are designed to directly bind to MHC-I molecules and stimulate CD8⁺ T cell responses, have poor serum stability, and are prone to rapid degradation. These are often conjugated to carrier proteins to enhance uptake and processing by APCs. In contrast, SLPs comprising 20 or more amino acids are more stable and immunogenic. They are efficiently processed by APCs and presented on both HLA-I and HLA-II molecules, resulting in stronger and broader immune responses. Notably, SLPs often outperform the full-length antigen from which they are derived in terms of immunogenicity [73].

To maximise the effectiveness of peptide vaccine mixtures, it is essential to achieve a balanced T cell response across all included epitopes. Otherwise, less immunogenic peptides may elicit suboptimal responses, potentially enabling tumour cells to escape immune detection. Enhancing immunogenicity can involve modifying peptides to increase their affinity for MHC-I molecules. One such approach is the design of analogue or heteroclitic peptides generated by substituting specific amino acids within the epitope sequence. These modified peptides have shown improved antigenicity and are capable of breaking immune tolerance by inducing more potent CD8⁺ T cell responses [74].

Moreover, the discovery of tumour antigens originating from non-coding genomic regions has expanded the repertoire of targets for cancer immunotherapy. Unlike mutation-derived neoantigens that are often patient-specific, cryptic antigens arising from the non-coding areas are potentially shared across individuals and may be directly linked to tumourigenesis. Their ability to mediate tumour rejection, as shown in murine models, highlights their clinical relevance [75].

Peptide-based cancer vaccines now undergoing clinical testing frequently include a straightforward mixture of antigenic peptides with TLR agonists, such as poly-ICLC, and standard adjuvants. The goal of next-generation peptide vaccines, on the other hand, is to increase efficacy by creating delivery mechanisms, including lipid-based carriers, that codeliver adjuvants and peptides to DCs, improving their antigen presentation and activation for T-cell priming. While reducing peripheral toxicity, these strategies target APCs in draining lymph nodes. Among these, lipid-nanoparticle formulations have demonstrated higher performance in preclinical investigations; nevertheless, complicated manufacturing procedures and challenges with large-scale, reliable production make clinical application difficult. One such vaccine formulation, in particular, showed noticeably higher efficiency than traditional adjuvants and was well tolerated in vivo [76].

### mRNA vaccines

mRNA-based therapeutics have emerged as a transformative drug class, revolutionizing treatment across infectious diseases and oncology [77]. Currently, two forms of mRNA are employed in vaccines: non-replicating mRNA, which encodes solely the target antigen, and self-amplifying mRNA (saRNA), which includes sequences for viral replication machinery, leading to extended and increased antigen expression along with stronger immune responses. Although both types are used in vaccines for infectious diseases, non-replicating mRNA is more frequently applied in cancer vaccine development [78].

mRNA cancer vaccines are capable of encoding TAA, TSA, and related cytokines, inducing both humoral and cellular immunity. These vaccines offer key advantages, including rapid and scalable production, design flexibility, relatively low cost, safety, absence of oncogenic potential, and robust immune activation. Importantly, mRNA vaccines do not integrate into the host genome, making them a promising and well-tolerated therapeutic option. Due to these reasons, mRNA vaccines are considered a formidable alternative to traditional vaccines [79].

**Figure 2:**
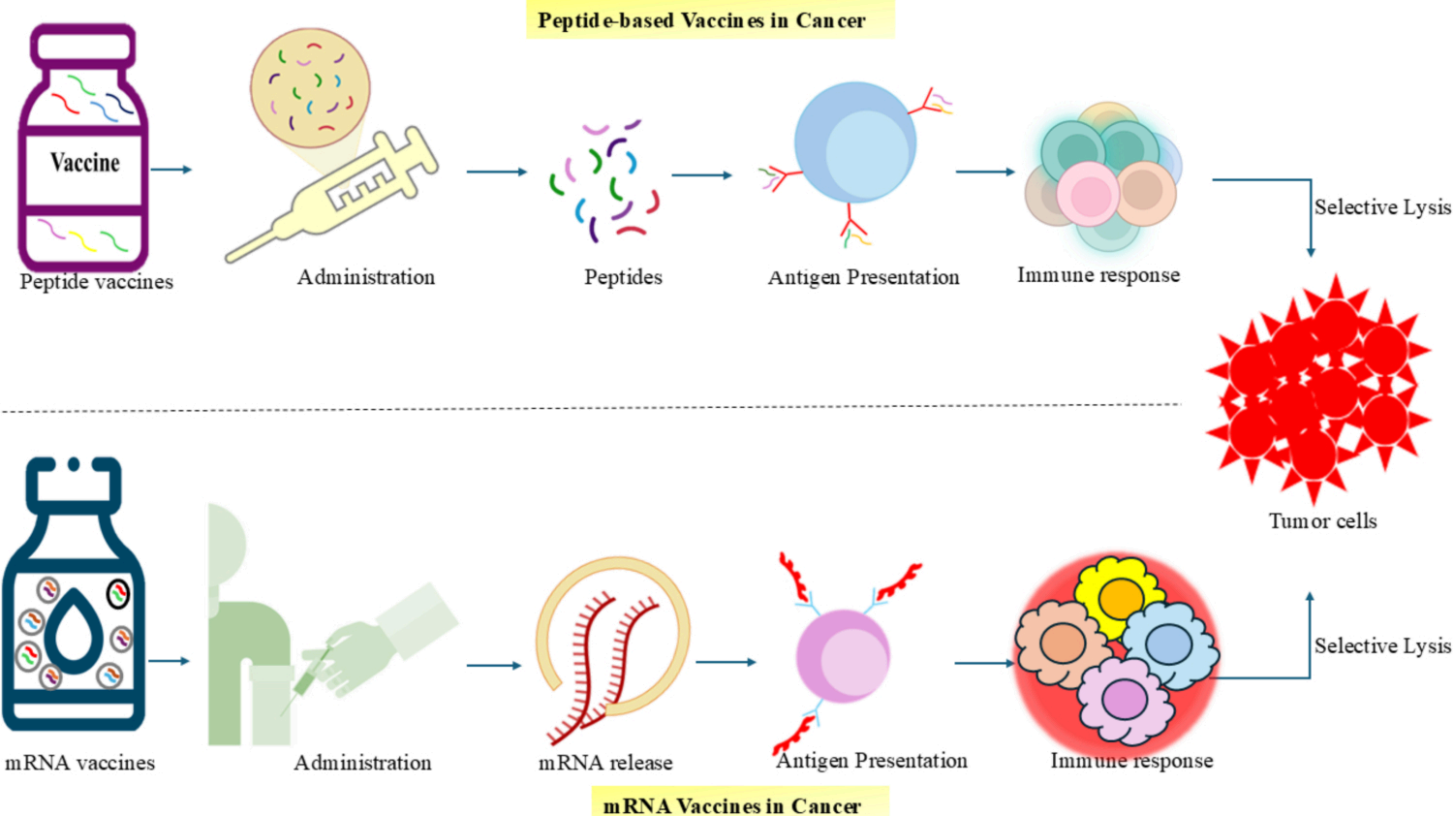
**Mechanisms of peptide and mRNA-based vaccines in cancer immunotherapy.** Peptide vaccines introduce tumour-specific peptides directly into the body, which are taken up by antigen-presenting cells (APCs) and presented via MHC molecules to activate an immune response targeting tumour cells. mRNA vaccines deliver synthetic mRNA encoding tumour antigens, which is translated into proteins inside APCs, followed by antigen presentation and immune activation. Both vaccine platforms aim to stimulate selective tumor cell destruction through cytotoxic immune responses. mRNA: messenger RNA; MHC: major histocompatibility complex.

Structural features of mRNA have been identified as immunostimulatory due to their interaction with innate immune receptors such as PKR, TLRs, and RIG-I, which is beneficial for vaccines encoding pathogen- or tumour-derived antigens. Antigen expression in conventional mRNA vaccines depends on the amount of mRNA delivered, often requiring large or repeated doses to achieve sufficient expression levels. Enhancing stability and systemic circulation is critical for efficacy, and this is achieved through synthetic modifications to prevent degradation, along with protective delivery systems using cationic lipids or polymer complexes [80]. The mechanism of Peptide and mRNA-Based Vaccines in Cancer Immunotherapy is depicted in Figure 2.

### DNA and viral vector vaccines

Viral vector vaccines utilize engineered viral particles to transport genes that encode specific target antigens into host cells. These vaccines are considered safe and effectively stimulate both innate and adaptive immune responses without introducing the entire pathogenic organism. Their flexibility in administration routes and inherent adjuvant properties through the expression of pathogen-associated molecular patterns (PAMPs) further enhance their immunogenicity. Viral vectors can also be engineered for tissue-specific antigen delivery and adjusted to be replication-competent or -deficient, improving safety and minimizing adverse reactions. By mimicking natural infection, they induce acute inflammation, activate immune detection, and support both mucosal and systemic immunity. In cancer therapy, viral vectors serve as ideal oncolytic viruses (OVs), capable of targeting tumour cells, releasing TAAs, and activating antitumour immune responses. Several OVs have already received approval, highlighting their therapeutic promise in cancer immunotherapy [81]. In preclinical and clinical studies, viral vectors have been used to test vaccines against infectious diseases like HIV, malaria, Ebola, and, more recently, SARS-CoV-2. Their ability to elicit robust antibody and cellular immune responses, both necessary for eradicating infected cells, gives them an advantage over conventional subunit vaccines. In addition, viral vectors can be designed for targeted antigen delivery, generate high immunogenicity without the need for adjuvants, and offer sustained protection (sometimes with a single dosage). To improve safety and decrease reactogenicity, the majority of viral vectors are genetically altered to lack the ability to replicate [82].

DNA vaccines are genetically engineered templates that transmit molecular instructions to trigger antigen-specific immune responses. They encode the target antigen and require effective delivery systems to express the corresponding protein and activate immunity. Generated through somatic mutations in cancer cells, neoantigens are novel peptides absent in normal tissues, rendering them suitable for targeted immune attacks. This concept has led to the rapid development of DNA vaccines aimed at neoantigen recognition. Key factors in their success include the careful selection of effective neoantigens and optimized delivery strategies to elicit precise and protective immune responses with minimal side effects. DNA vaccines are not subject to the risks of virulence reversion seen in live attenuated vaccines or the side effects of inactivated ones [83].

DNA vaccines provide versatile design capabilities and can be engineered to encode one or multiple antigens within a single plasmid, allowing protection against a range of diseases. Notably, they stimulate both humoral (antibody-mediated) and cellular (T-cell-mediated) immune responses, with cellular immunity playing a particularly vital role in antitumour activity [83]. As an emerging approach in cancer immunotherapy, DNA vaccines can activate innate immune mechanisms and, depending on their formulation and delivery method, induce targeted humoral and cellular immune responses. They are easy to engineer, rapidly producible in large amounts, and highly stable for storage and transport. Unlike live attenuated vaccines, they pose no infection risk and do not induce anti-vector neutralizing antibodies, allowing for multiple dosing [84].

Compared to previous cancer vaccination strategies, DNA vaccines offer distinct benefits. They are simple to produce in large quantities and provide a long-lasting immunological memory, which lowers the chance of recurrence. They are less expensive, easier to carry, and more stable, but do require storage in freezing temperatures, unlike mRNA vaccines. DNA vaccines are safe and generally well-tolerated; only mild autoimmune reactions are infrequently observed, and they may be customized for each patient. They do not carry the same risk of pathogenic infection as viral vectors, and repeated dosages do not result in the production of neutralizing antibodies; thus, they can be administered more than once without losing their effectiveness. DNA vaccines, instead of mRNA cancer vaccines, provide sustained antigen expression, maintaining immune responses and possibly lowering the number of doses needed [85].

### DC vaccines

Multiple studies indicate that eliciting a robust and long-lasting cytotoxic T-cell response necessitates either the concurrent or sequential activation of different types of APC subsets, particularly DCs and macrophages. Among APCs, DC vaccines targeting tumour antigens represent a promising approach in immunotherapy, aiming to enhance the patient’s immune response against their tumour [86].

DCs are the most potent and specialized APCs, essential for initiating and orchestrating both innate and adaptive immune responses. They represent a diverse group of cells, typically classified by their developmental origin into cDC1 and cDC2, plasmacytoid DCs (pDCs), and monocyte-derived DCs (MoDCs). Advances in high-throughput single-cell analysis have recently revealed additional subsets and states, such as DC3, deepening our understanding of their functions and developmental pathways. Due to their specialized function in inducing and controlling antigen-specific immune reactions and immune tolerance, DCs are invaluable in the field of vaccine development. Harnessing their diverse antigen-presenting abilities holds significant potential for improving antitumour responses induced by therapeutic vaccines. While DC vaccines have shown limited efficacy when used alone for solid and hematologic tumours, their combination with other anticancer therapies offers renewed hope for orchestrating a targeted antitumour T-cell response [87].

DCs are capable of presenting antigens to both CD4^+^ and CD8^+^ T-cells, thereby activating humoral and cellular branches of adaptive immunity. As such, DC vaccines also serve as safe adjuncts to multimodal cancer therapies, potentially enhancing the effects of chemotherapy and immunotherapies like immune checkpoint inhibitors. DC vaccines are generally classified into three generations. First-generation DC vaccines utilized either natural DCs isolated from patients or ex vivo-generated MoDCs that were not further matured. Second-generation vaccines utilized fully matured MoDCs, generated using specific maturation cocktails, and loaded with tumour antigens in the form of recombinant or synthetic peptides or tumour cell lysates obtained through physical or mechanical disruption methods such as necrosis. To ensure total elimination of cancer cells, these lysates were often subjected to additional treatments like irradiation (UV, X-ray, or gamma radiation) and/or heat shock, a process known as avitalization, which also aimed to enhance immunogenicity through the induction of specific immune-stimulating effects [88].

Compared to conventional vaccine modalities, DC vaccines provide several benefits. DC vaccines produce robust antibody and CTL responses, which are essential for eradicating tumour or pathogen-infected cells, in contrast to other vaccinations that could mainly produce humoral immunity. To improve the accuracy of the immune response, DC vaccines can also be customized by adding patient-specific antigens, such as neoantigens [87].

### Personalized neoantigen vaccines

Personalized tumour neoantigen vaccines have made substantial strides in clinical trials, showing encouraging outcomes across multiple cancer types. They have also continued to advance in combination with other immunotherapies [89]. Neoantigen-based vaccines offer several advantages over traditional TAA-based approaches. Unlike TAAs, which are often self-antigens re-expressed or overexpressed in tumours but still present in normal tissues, neoantigens are uniquely derived from tumour-specific somatic mutations, gene rearrangements, or alternative splicing events. Their absence in normal tissues enhances their tumour specificity, minimizing off-target effects [90, 91].

Since neoantigens are identified as foreign by the immune system, they evade both central and peripheral tolerance mechanisms, allowing for the generation of strong antitumour immune responses. Personalized neoantigen vaccines leverage this immunogenic potential to activate tumour-specific T cells, leading to effective tumour shrinkage and the possibility of long-lasting immune memory [90].

**Figure 3:**
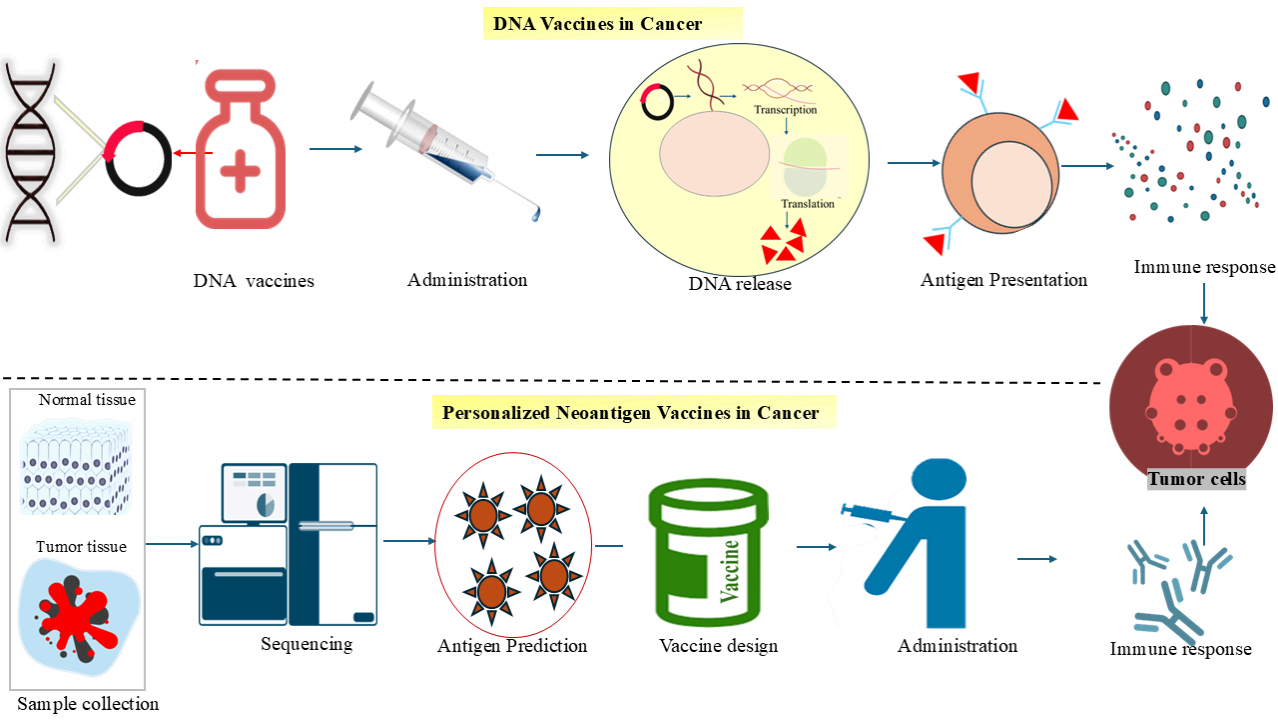
**Mechanisms of DNA-based and personalized neoantigen vaccines in cancer immunotherapy.** The mechanism of DNA vaccines involves administering plasmid DNA to the patient, which leads to the intracellular transcription and translation of tumour antigens. This is followed by antigen presentation by immune cells and the activation of tumour-specific immune responses. Next, a personalized neoantigen vaccine is created, where normal and tumour tissue samples are collected from the patient and sequenced to identify tumour-specific mutations. Computational tools predict immunogenic neoantigens, which are used to design patient-specific vaccines. After administration, the immune system mounts a targeted response against tumour cells based on the individual’s unique antigen profile.

Neoantigen immunotherapy represents a fully personalized approach, as the relevant mutations differ between individuals. These vaccines target tumours based on their unique mutational signatures rather than shared oncogenic pathways, offering a precise mechanism for immune activation [91]. TAAs, which are expressed on both tumour and normal cells, are the main target of conventional tumour vaccines. Neoantigen-based vaccines, on the other hand, have significant benefits, such as increased specificity and fewer adverse effects. However, they frequently fall short of producing potent or enduring therapeutic effects when used as monotherapy. More effective anticancer responses can be obtained by combining neoantigen vaccinations with other approaches such as immune checkpoint inhibition or TME modification [92]. Consequently, neoantigen vaccines provide a robust and accurate strategy for cancer treatment, enhancing both efficacy and long-term protection against recurrence. Mechanisms of DNA-based and personalized neoantigen vaccines in cancer immunotherapy are depicted in Figure 3.

## Challenges and limitations

Immunogenicity introduces additional risks by potentially triggering immune responses that compromise the effectiveness of vaccine-based therapies. A major hurdle is the selection of appropriate targets, particularly when treating solid tumours, where physical barriers and tumour heterogeneity hinder therapeutic penetration and efficacy. The multiplicity of tumour-associated targets further complicates predictions of the therapeutic window and elevates the risk of off-target effects and toxicity, necessitating rigorous design strategies and extensive preclinical testing to ensure safety and efficacy [93].

Detecting non-canonical peptides poses a distinct challenge because of their limited length and typically low abundance, making them difficult to detect through conventional shotgun MS. Before the integration of Ribo-seq, MS-based peptide identification relied heavily on reference databases, which are often insufficient for detecting peptides derived from unconventional sources. In silico translation of entire transcriptomes increases database size exponentially, leading to a high rate of false positives. Thus, optimization strategies have been developed to manage database size more effectively and improve the specificity of MS-based identification of non-canonical peptides [39].

Moreover, the diversity of MAPs adds to the complexity of identification, as these include peptides originating from atypical transcriptional and translational events. Such sources include alternative splicing, frameshifts, and translation from non-coding RNAs, UTRs, introns, intergenic regions, and antisense RNAs [94]. Tumour heterogeneity further complicates this landscape. Deficient mismatch repair (dMMR) and microsatellite instability (MSI) are key drivers of ITH, which encompasses a broad range of molecular and cellular disparities within tumours, including genomic instability, epigenetic alterations, dysregulated gene expression, PTMs, and diverse immune microenvironments. These variations can bias the assessment of immunotherapy biomarkers such as PD-1, tumour mutational burden (TMB), and dMMR/MSI, leading to inconsistent clinical outcomes. To address this, therapeutic strategies must consider both spatial and temporal heterogeneity to effectively guide patient-specific treatment approaches [89].

Cancer cells also employ mechanisms to evade immune surveillance by shaping the TME from the early stages of oncogenesis. This immunosuppressive environment hinders the immune system’s ability to detect and destroy tumour cells, allowing malignant growth to outpace host defense mechanisms. The immune response, which unfolds in phases of recognition, processing, and reaction, is often subverted by the cancerous microenvironment, diminishing the effectiveness of immunotherapies [95].

In addition, the production of personalized or cell-based cancer vaccines brings logistical and economic challenges. The complexity of manufacturing processes, long production timelines, and the high costs associated with potency validation and batch release testing can delay treatment availability. Such delays are particularly critical for patients with progressive disease, who may deteriorate while awaiting access to these novel therapies [96].

Although traditional vaccines have saved many lives, they have several drawbacks, such as sluggish manufacture, high costs, and the requirement for specialized facilities. Influenza vaccinations, for instance, need viral propagation in eggs, which makes it challenging to increase production during pandemics quickly. Furthermore, live attenuated vaccines must be stored strictly in a cold chain, which is challenging to maintain in environments with limited resources and may result in deterioration and decreased efficacy. By facilitating quick development, scalable manufacturing, and potent immune responses without the use of live pathogens, new vaccine platforms such as mRNA and DNA vaccines get around many of these limitations. With benefits in safety, effectiveness, and production efficiency, mRNA vaccines give host cells genetic instructions to manufacture viral antigens. Significant developments that improved the effectiveness of COVID-19 mRNA vaccines included nucleoside modification and ionizable lipid nanoparticles. Additionally, these technologies allow for customized cancer vaccinations that target mutations unique to each patient. Nucleic acid vaccines hold promise against new threats, for example, the Marburg virus (MARV), for which there are currently no licensed treatments or vaccinations. Rapid genome sequencing and scalable manufacturing have transformed pandemic preparedness, enabling quicker and more efficient reactions to medical emergencies [97].

Compounding these issues, the clinical trial ecosystem essential for bringing such therapies to market is itself under strain. The multi-phase nature of trials involves numerous stakeholders, including investigators, sponsors, regulatory bodies, and patient advocacy groups. However, systemic inefficiencies, staffing shortages, and outdated operational models have hindered trial execution and slowed the development of innovative therapies. The disconnection from a patient- and community-centred approach further threatens equitable access to clinical trials and may jeopardize future progress in cancer drug development [98].

Together, these interconnected challenges from peptide identification and tumour heterogeneity to logistical, economic, and systemic barriers pose significant threats to the advancement and implementation of cancer vaccine immunotherapy.

## Conclusions

Cryptic and non-canonical antigens represent a paradigm shift in cancer vaccine development, offering novel, tumour-specific targets that can overcome the limitations of traditional TAAs. These unconventional antigens, derived from previously overlooked regions of the genome or produced through atypical molecular mechanisms, are capable of eliciting robust immune responses with minimal risk of autoimmunity. Leveraging cutting-edge technologies such as Ribo-seq and immunopeptidomics has expanded our understanding of the immunogenic landscape and accelerated the identification of these targets. While various vaccine platforms have shown promise in delivering non-canonical antigens, translating these discoveries into effective and scalable therapies will require overcoming key challenges, including immunosuppressive TMEs, antigen variability, and production complexities. Future success hinges on integrating personalized approaches with advanced delivery systems and combination therapies, ultimately driving forward the precision and efficacy of cancer immunotherapy.

## Abbreviations

AI: artificial intelligence
APCs: antigen-presenting cells
cDC1: conventional dendritic cell 1
CTL: cytotoxic T lymphocyte
DCs: dendritic cells
dMMR: deficient mismatch repair
ER: endoplasmic reticulum
HLA: human leukocyte antigen
ITH: intratumoural heterogeneity
lncRNAs: long non-coding RNAs
MHC-I: major histocompatibility complex class I
MoDCs: monocyte-derived dendritic cells
mRNA: messenger RNA
MS: mass spectrometry
MSI: microsatellite instability
ncMAPs: non-canonical major histocompatibility complex-associated peptides
ncORFs: non-canonical open reading frames
ORF: open reading frame
OVs: oncolytic viruses
PCPS: proteasome-catalyzed peptide splicing
pre-mRNA: precursor messenger RNA
PTMs: post-translational modifications
Ribo-seq: ribosome profiling
RNA-seq: RNA sequencing
SLPs: synthetic long peptides
sORFs: small open reading frames
TAAs: tumour-associated antigens
TLR: Toll-like receptor
TME: tumour microenvironment
TSAs: tumour-specific antigens
uORFs: upstream open reading frames
UTRs: untranslated regions
WES: whole exome sequencing
WGS: whole genome sequencing

## Declarations

### Copyright

© The Author(s) 2025.
